# Oxcarbazepine-induced hyponatremia: A case report

**DOI:** 10.1192/j.eurpsy.2021.1286

**Published:** 2021-08-13

**Authors:** M. Barbosa Pinto, M. Viseu De Carvalho, F. Gomes Tavares, J. Reis

**Affiliations:** Psychiatry, Centro Hospitalar Universitário do Algarve - Unidade Faro, Faro, Portugal

**Keywords:** Oxcarbazepine, Hyponatremia, adverse effects

## Abstract

**Introduction:**

Oxcarbazepine (OXC) is an antiepileptic drug widely used in the treatment of bipolar disorder (BD), specially when there are side effects with other mood stabilizers. Nevertheless, it isn’t innocuous of adverse effects and its consequences can even endanger the patient’s life.

**Objectives:**

Brief review of the literature on OXC-induced hyponatremia and exposure of a case report.

**Methods:**

Review of the literature through research in the PubMed database, using the following keywords: “oxcarbazepine”, “hyponatremia” and “adverse effects”.

**Results:**

Although most of the patients are asymptomatic, hyponatremia is one of the most important side effects of OXC. About 29.9% of the patients develop hyponatremia, but only 2.5-3% of psychiatric patients develop severe hyponatremia. The risk of hyponatremia is higher during the first three months of treatment. Severe and/or symptomatic hyponatremia has important clinical implications and may be associated with neurological damage, including seizures, brain stem herniation and death. A 44-year-old woman diagnosed with BD started OXC due to drug intoxications with other mood stabilizers. Six days after initiating treatment, she presented persistent vomiting and severe hyponatremia was detected in blood tests. OXC was suspended with symptomatic resolution.

**Conclusions:**

Healthcare professionals should be alert to symptoms that may arise in patients under OXC. Periodic evaluations of serum sodium levels should be carried out. Cases of severe and/or symptomatic hyponatremia should be rapidly identified and treated in order to reduce the risk of developing brain injury and death.

